# Treatment of canine atopic dermatitis: 2015 updated guidelines from the International Committee on Allergic Diseases of Animals (ICADA)

**DOI:** 10.1186/s12917-015-0514-6

**Published:** 2015-08-16

**Authors:** Thierry Olivry, Douglas J. DeBoer, Claude Favrot, Hilary A. Jackson, Ralf S. Mueller, Tim Nuttall, Pascal Prélaud, for the International Committee on Allergic Diseases of Animals

**Affiliations:** Department of Clinical Sciences, College of Veterinary Medicine, North Carolina State University, 1060 William Moore Drive, Raleigh, 27606 NC USA; Department of Medical Sciences, School of Veterinary Medicine, University of Wisconsin, 2015 Linden Drive, Madison, 53706 WI USA; Clinic for Small Animal Internal Medicine, Dermatology Department, Vetsuisse Faculty, University of Zürich, Winterthurerstrasse 260, 8057 Zürich, Switzerland; Dermatology Referral Services LTD, 528 Paisley Road West, Glasgow, Scotland G51 1RN UK; Medizinische Kleintierklinik, Centre for Clinical Veterinary Medicine, Ludwig-Maximilian University, Veterinärstrasse 13, 80539 Munich, Germany; Royal (Dick) School of Veterinary Studies, The University of Edinburgh, Easter Bush Campus, Roslin, Scotland EH25 9RG UK; Clinique Advetia, 5 rue Dubrunfaut, Paris, 75012 France

**Keywords:** Atopic dermatitis, Canine, Dogs, Evidence-based medicine, Guidelines, Treatment

## Abstract

**Background:**

In 2010, the International Task Force on Canine Atopic Dermatitis (now International Committee on Allergic Diseases of Animals, ICADA) published the first consensus guidelines for the treatment of atopic dermatitis (AD) in dogs. This is the first 5-year minor update of this document.

**Results:**

The treatment of acute flares of AD should involve the search for, and then elimination of, the cause of the flares, bathing with mild shampoos, and controlling pruritus and skin lesions with interventions that include topical and/or oral glucocorticoids or oclacitinib. For chronic canine AD, the first steps in management are the identification and avoidance of flare factors, as well as ensuring that there is adequate skin and coat hygiene and care; this might include more frequent bathing and possibly increasing essential fatty acid intake. The medications currently most effective in reducing chronic pruritus and skin lesions are topical and oral glucocorticoids, oral ciclosporin, oral oclacitinib, and, where available, injectable recombinant interferons. Allergen-specific immunotherapy and proactive intermittent topical glucocorticoid applications are the only interventions likely to prevent or delay the recurrence of flares of AD.

**Conclusions:**

This first 5-year minor update of the international consensus guidelines for treatment of AD in dogs further establishes that the treatment of this disease is multifaceted, and that interventions should be combined for a proven (or likely) optimal benefit. Importantly, treatment plans are likely to vary between dogs and, for the same dog, between times when the disease is at different stages.

**Electronic supplementary material:**

The online version of this article (doi:10.1186/s12917-015-0514-6) contains supplementary material, which is available to authorized users.

## Background

In 2010, the International Task Force on Canine Atopic Dermatitis (ITFCAD), now International Committee on Allergic Diseases of Animals (ICADA; www.icada.org) generated the first guidelines for treatment of atopic dermatitis (AD) in dogs [1]. These recommendations, published in English and translated in 17 other languages, were designed and made freely downloadable for a global general practitioner audience. While new drugs have become available in the last 5 years, others are no longer so, and therapeutic regimens have continued to evolve. For these reasons, the ICADA membership decided to update these guidelines on a 5-year basis. While complete rewrites are planned to occur every 10 years, minor updates are to be written 5 years into each decade; this is the first quinquennial minor rewrite to the 2010 canine AD treatment guidelines [1].

As for the first version of these directives, readers should remember several basic principles that underlie this document:

1. Recommendations are generally made from evidence derived from previously published randomized controlled trials (RCTs) and systematic reviews [2–4]. Practitioners must keep in mind that statistically significant changes in trial’s outcome measures do not imply that the intervention will be effective in all patients, or that the owners will be satisfied with the recommended product. Moreover, clinical trials generally test the efficacy of a single intervention, while, in daily practice, the best clinical benefit normally requires the combination of multiple treatments. Consequently, results of clinical trials will usually underestimate the synergistic potential of the tested drug when it is included in a multi-intervention treatment protocol.
2. In multiple sections of these guidelines, readers will find that there is a lack of or insufficient evidence supporting the efficacy of a specific intervention. Such statement does not mean that the discussed intervention will not be effective in their patient, but rather that it has not been tested sufficiently to assess if it offers any benefit.
3. As in the first version of these guidelines, when recommendations are made for an intervention supported by one or more trials done with a specific product, we mention the generic drug name followed by the brand and company indicated in the paper reporting the study results. In all other cases, recommendations only provide generic drug names. Importantly, the recommendation for a specific product does not imply the endorsement of the product or its maker by the ICADA. A recommendation only means that at least one clinical trial exists that suggests the drug’s benefit, or, in the absence of such trial, that there is consensus among authors for recommending this intervention.
4. Finally, and as done previously, this update is divided into three different sections: recommendations for i) the management of acute flares of canine AD, ii) the treatment of chronic skin lesions of AD, and, iii) the interventions to prevent disease relapses. For typical case scenarios that could benefit from these recommendations, the readers are referred to the 2010 version of these guidelines [1]. In each section, treatment options are listed in a particular order. By no means do we imply that all interventions are recommended—or even needed—for each patient in that very same order. Recommendations must be evaluated by veterinarians taking in consideration their unique patient and pet owners. Practitioners should always assess the benefit, side effects, practicability, cost and availability of the proposed treatments, which often will have to be combined for an optimal outcome.

This paper is aimed at being a shorter update of the longer original version of the guidelines [1]. Each section will contain an abbreviated summary of the 2010 recommendations, followed by a “2015 update” with supporting information for the proposed change or update. Supporting data published in the 2010 guidelines will normally not be repeated. In each section, we will clearly state where there was no obvious need for updating the 2010 recommendations.

Importantly, the authors decided to change the strength of recommendation (SOR) and category of evidence (COE) grading schemes used in the 2010 guidelines to the simplified and less confusing SORT scoring system (Table 1) [5]. As before, an SOR of lower alphabetic order and a quality of evidence (QOE) of lower Roman numeral should be considered of greater value than those with higher letters and numbers. However, readers should not attempt to compare the SORs and COEs/QOEs between the 2010 and 2015 versions of these guidelines, are these scores are not designed to be transposable.

**Table 1:** Strength of recommendation taxonomy (SORT)

Strength of recommendation (SOR)
A = based on consistent and good quality patient-oriented evidence
B = based on inconsistent or limited quality patient-oriented evidence
C = based on consensus, usual practice, opinion, disease-oriented evidence or case series
Quality of the evidence (QOE):
1 = good quality, patient-oriented
2 = limited quality, patient-oriented
3 = other evidence (usual practice, opinion, disease oriented evidence)

Furthermore, in this update, and to facilitate the comparison between this and future versions of the guidelines, each section will be numbered.

Finally, and as done previously, we provided, as an online document, a one-page summary of the recommendations developed herein (Additional file 1).

### A. Treatment of acute flares of AD

This section is relevant to the treatment of dogs with case scenarios 1a and 1b described in the 2010 version of these guidelines [1]; these can be accessed freely on the following site: http://onlinelibrary.wiley.com/doi/10.1111/j.1365-3164.2010.00889.x/suppinfo.

#### A.1. Identification and avoidance of flare factors

- *A.1.a. Identification and removal of allergenic causes of flares*
  - Summary of the 2010 guidelines:
    - Recognized allergenic causes of acute flares of canine AD are a recent increased exposure to environment allergens (especially house dust mites and pollens), the ingestion of food ingredients, and flea or other insect bites. Flares will normally only occur if the dog is hypersensitive to these different allergens and if the allergen load is sufficiently high to trigger flares. The identification and, if at all possible, the elimination of contact with, or ingestion of, such allergens are important to prevent further worsening or recurrences of the flares [1].
  - Updated 2015 recommendations:
    - There are no proposed changes to the 2010 recommendations (SOR C).

- *A.1.b. Evaluation of use of antimicrobial therapy*
  - Summary of the 2010 guidelines:
    - Bacterial and yeast skin and ear infections are common causes of flares in dogs with AD. The treatment of such infections usually consists of topical and/or systemic antimicrobials [1].
  - Updated 2015 recommendations:
    - There are no major changes to the 2010 recommendations (SOR C). To improve efficacy and antimicrobial stewardship, veterinarians are advised to follow antimicrobial treatment guidelines established in their country of practice and/or in international consensus recommendations (SOR C) [6, 7]. Importantly, veterinarians and pet owners should watch for a drying or irritating effect of topical antimicrobials—especially shampoos—that might induce a flare of AD in their patient (SOR C).

#### A.2. Improvement of skin and coat hygiene and care

- *A.2.a. Bathing with a non-irritating shampoo*
  - Summary of the 2010 guidelines:
    - Bathing with an emollient shampoo containing lipids, complex sugars and antiseptics (Allermyl, Virbac) has been shown to have a modest and short-lived antipruritic effect. Other topical emollients have not been proven to reduce pruritus. The intensity and frequency of bathing may be the most important factors to relieve itch [1].
  - Updated 2015 recommendations:
    - Emollient formulations containing either lipids, complex sugars and antiseptics (Allermyl, Virbac) or phytosphingosine, raspberry oil and lipids (Douxo Calm, Ceva) have been shown to provide a modest effect on skin lesions and pruritus in allergic dogs (SOR B); this benefit is likely highest in dogs with mild AD (SOR C). The intensity and frequency of bathing may be the most important factor in relieving pruritus (SOR B). Other topical emollients have not been proven to consistently reduce signs of AD in dogs (SOR C).
  - Basis for the updated recommendations:
    - A recent three-week small RCT revealed the nearly equivalent reduction of skin lesions and pruritus in allergic dogs using either Allermyl shampoo or a Douxo Calm shampoo and foam combination (QOE 2) [8]. These results mirror those from a previous small trial employing Allermyl, Douxo Calm shampoo or a Douxo Calm shampoo and spray regimen (QOE 2) [9].

#### A.3. Reduction of pruritus and skin lesions with pharmacological agents

- *A.3.a. Short-term treatment with topical glucocorticoids*
  - Summary of the 2010 guidelines:
    - Topical glucocorticoid sprays are effective for the treatment of acute flares of canine AD. Such intervention is especially suitable for localized skin lesions and for short durations. Treatment duration and frequency should be tailored to the patients’ clinical signs [1].
  - Updated 2015 recommendations:
    - Topical glucocorticoid sprays (Cortavance, Virbac [SOR A]; Genesis, Virbac US [SOR B]) are effective for treatment of flares of canine AD. In the absence of availability of these formulations, other topical glucocorticoid formulations are theoretically likely to be beneficial, but the efficacy and safety of these medications will vary with the strength of glucocorticoid and vehicle used (SOR C). Topical glucocorticoids are especially beneficial for localized skin lesions and for short durations; care must be taken to avoid the steroid-induced skin atrophy that will nearly always develop after long-term daily application of the product at the same skin sites (SOR C). Treatment duration and frequency of use should be tailored to each patient; applications should normally continue until complete and stable remission of signs (SOR C).
  - Basis for the updated recommendations:
    - In addition to the previously available clinical trial data, a small study confirmed that a one to two-week daily application of an hydrocortisone aceponate spray (Cortavance, Virbac) significantly improved lesions and pruritus in atopic dogs (QOE 2) [10].

- *A.3.b. Short course of oral glucocorticoids or oclacitinib*
  - Summary of the 2010 guidelines:
    - Oral prednisolone, prednisone or methylprednisolone at 0.5 mg/kg once to twice daily improve clinical signs of dogs with severe or extensive AD. Side effects of oral glucocorticoids are generally proportional to drug potency, dosage and duration of administration. The treatment of acute flares of canine AD with long-acting injectable glucocorticoids is not recommended. Because most dogs with AD have signs that respond to oral glucocorticoids, failure of rapid clinical benefit with this intervention should prompt clinicians to reconsider alternative diagnoses or the presence of secondary complications (for example, skin infections, ectoparasitism or other nonatopic food reactions) [1].
  - Updated 2015 recommendations
    - Oral prednisolone, prednisone or methylprednisolone given at 0.5 to 1.0 mg/kg per day, in one or divided into two doses, is likely to improve clinical signs of dogs with severe or extensive AD (SOR A). Adverse effects of oral glucocorticoids are normally proportional to drug potency, dosage and duration of administration. The treatment of acute flares of canine AD with long-acting injectable glucocorticoids is not recommended (SOR C).
    - Oclacitinib (Apoquel, Zoetis) can be prescribed at 0.4–0.6 mg/kg orally twice daily for up to 14 days to rapidly reduce skin lesions and pruritus in dogs with AD (SOR A). Short-term treatment with oclacitinib appears safe.
    - Because of theoretical concerns for a potential dose-dependent drug-induced immunosuppression, the concomitant use of oral glucocorticoids with oclacitinib is likely contraindicated, especially in case of infections, though such combined use has not been evaluated (SOR C).
    - As most signs of canine AD are expected to respond to oral glucocorticoids or oclacitinib, clinicians should reconsider alternative diagnoses and/or the presence of secondary complications (for example, skin infections, ectoparasitism, nonatopic food reactions etc…) if there is no rapid clinical benefit after treating atopic dogs with these drugs (SOR C).
  - Basis for such recommendations:
    - Additional studies, which used prednisone or prednisolone as positive treatment controls for comparison with oclacitinib (QOE 1) [11] or ciclosporin (QOE 2) [12, 13], have confirmed the rapid efficacy of oral glucocorticoids for treatment of canine AD. Oclacitinib has been shown to reduce pruritus and clinical signs significantly better than placebo (QOE 1) [14] and as well as—or, at the 14 day time point, better than—prednisolone (QOE 1) [11]. Short-term adverse effects of oclacitinib appear minor.

- *A.3.c. Interventions likely to be of little or no benefit to treat acute flares of canine AD*
  - *A.3.c.1. Antihistamines*
    - Summary of the 2010 guidelines:
      - Type-1 antihistamines (i.e. H1 histamine receptor antagonists) are not likely to be beneficial after a flare of AD has occurred. There is no conclusive evidence for the efficacy for type 1 antihistamines for treatment of active AD in dogs [1].
    - Updated 2015 recommendations:
      - Oral type 1 antihistamines might provide a small and limited benefit in some dogs with AD (SOR B). Due to their mode of action and for an optimal benefit, oral type 1 antihistamines should preferably be given before a flare occurs to block the effects of histamine (SOR C). Clinical benefit might also occur due to the sedative effect of first generation type 1 antihistamines (e.g. diphenhydramine, chlorpheniramine…) (SOR C). Due to their limited efficacy, type 1 antihistamines are likely to be more beneficial in dogs with mild AD (SOR C). There is no evidence supporting the use of topical type 1 antihistamine formulations to treat canine AD (SOR C).
    - Basis for such recommendations:
      - Approximately 25 % of clients that gave oral antihistamines to their atopic dogs reported these to be at least very effective in a retrospective survey (QOE 2) [15]. An RCT reported that two oral antihistamines, a hydroxyzine and chlorpheniramine combination (Histacalmine, Virbac) and dimetindene (Fenistil, Novartis), mildly improved pruritus and skin lesions in dogs with AD (QOE 2) [16]. In contrast, the administration of an oral type 1 antihistamine (hydroxyzine) did not prevent the development of skin lesions in an experimental model of acute AD in house dust mite-sensitized dogs (QOE 3) [17].

- *A.3.c.2. Essential fatty acids (EFAs)*
  - Summary of 2010 guidelines:
    - Oral EFAs are not useful to treat acute flares of AD due to the length of time needed for any possible beneficial effect to occur [1].
  - Updated 2015 recommendations:
    - There are no proposed changes to the 2010 recommendations (SOR C).
  - Basis for such recommendations:
    - A systematic review identified no additional evidence supporting the effectiveness of oral EFA supplementation for treatment of acute flares since the publication of the 2010 guidelines [4]. A small RCT testing a topical lipid complex containing EFAs (Allerderm Spot-on, Virbac) did not show an effect in reducing skin or pruritus two weeks after application. As a result, this formulation is unlikely to offer any benefit in the management of acute flares of canine AD (QOE 2) [18].

- *A.3.c.3. Calcineurin inhibitors*

- Summary of 2010 guidelines:
  - The slow onset of action of topical (e.g. tacrolimus) and oral (e.g. ciclosporin) calcineurin inhibitors makes them unsuitable for managing acute flares of AD [1].
- Updated 2015 recommendations:
  - There are no proposed changes to the 2010 recommendations (SOR C).

### B. Treatment of chronic canine AD

This section is relevant to the treatment of dogs with case scenarios 2a and 2b described in the 2010 version of these guidelines [1]; these can be accessed freely on the following site: http://onlinelibrary.wiley.com/doi/10.1111/j.1365-3164.2010.00889.x/suppinfo.

#### B.1. Identification and avoidance of flare factors

- *B.1.a. Performance of dietary restriction-provocation trials in dogs with nonseasonal AD*
  - Summary of the 2010 guidelines:
    - Dogs with adverse food reactions can present with clinical signs of AD, and some dogs exhibit concurrent allergy to environmental and food allergens. Restriction-provocation dietary trials are the standard method to diagnose food-induced AD. Clinicians should consider repeating dietary trials in dogs with a previously well-controlled AD that is now relapsing [1].
  - Updated 2015 recommendations:
    - Overall, there are no major changes to the 2010 recommendations (SOR C).
    - In dogs, as in humans, food allergy can manifest with clinical signs of AD or other syndromes (e.g. urticaria or others) (SOR C) [19]. The current gold standard for the diagnosis of food allergy remains a restriction trial with novel and/or hydrolysed diets followed by provocation with original food items once signs have abated during the restriction phase (SOR C). An 8-week restriction-provocation dietary trial should permit the diagnosis of food allergy in most dogs (SOR A). In case of dubious response to the first food change, additional dietary trials may be needed, especially if: 1) the history suggests an inappropriate diet selection (e.g. lack of “novelty” of ingredients or over-the-counter ingredient diets, as opposed to those designed for veterinarian prescription) for the first trial, or 2) the dogs present with perianal pruritus and/or associated gastro-intestinal signs, or 3) previously well-controlled atopic dogs experience a flare that cannot be controlled by means that were helpful before (SOR C).
    - It is speculated that the presence of storage mites in dry dog foods might cause some relapses of AD because of their allergenic crossreactivity with house dust mites to which atopic dogs are frequently hypersensitive (SOR C). However, there is currently no evidence suggesting that avoiding dry commercial dog foods is beneficial in dogs hypersensitive to storage and/or house dust mites (SOR C). Freezing dry dog foods might reduce contamination with storage mites, but the impact of such freezing on the clinical signs of mite-hypersensitive dogs is unknown (SOR C). Nevertheless, to decrease excessive storage mite contamination, owners should be encouraged to avoid storing dry dog foods in humid and warm areas, and they should be advised to store foods in clean and sealed containers (SOR C).
  - Basis for such recommendations:
    - A recent critically-appraised topic established that an 8-week elimination diet should lead to a remission of signs in more than 90 % of dogs with cutaneous adverse food reactions (QOE 1) [20].
    - Three studies have demonstrated that “non-prescription” pet foods obtained from pet stores or other retail channels (including foods supposedly containing limited ingredients) frequently contain traces of ingredients that are not listed on the label [21–23]. Whether or not such contamination would induce flares in dogs with food-induced AD is not known.
    - Two thirds of dogs with concurrent AD and food allergies exhibit perianal pruritus (QOE 2) [24].
    - House dust and storage mites and faeces are rarely present in commercial dry dog foods (QOE 3) [25, 26]. Storage of foods in paper bags (QOE 3) [25, 26], and especially in environmental conditions of moderate temperatures and high humidity, increases *Tyrophagus* storage mite numbers (QOE 3) [26]. Nevertheless, the concentration of mite allergens on the floor adjacent to stored dog food bags appears much higher than in the food itself (QOE 3) [25].

- *B.1.b. Implementation of a flea control regimen*
  - Summary of the 2010 guidelines:
    - Dogs with AD should be treated year-round with an effective flea control regimen. Systemic and oral adulticides are recommended in case of repeated shampooing to prevent the wash off of topical flea control products [1].
  - Updated 2015 recommendations:
    - There are no changes to the 2010 recommendations (SOR C). Insecticides that demonstrate long effect and fast residual speed of kill should be theoretically more effective in dogs with AD that are hypersensitive to flea bites (SOR C).
  - Basis for such recommendations:
    - A trial established the superiority of spinosad (Comfortis, Elanco) over a fipronil/(S) methoprene combination (Frontline Plus, Merck) in the control of flea-associated pruritus in field conditions; the higher efficacy of spinosad could be due to its prolonged activity and/or fast residual speed of kill (QOE 2) [27].

- *B.1.c. Performance of allergen-specific intradermal and/or IgE serological tests to identify possible allergenic flare factors*
  - Summary of the 2010 guidelines:
    - Allergen-specific intradermal testing (IDT) and/or IgE serologies are helpful to identify hypersensitivity to environmental allergens in dogs with AD. Positive immediate IDT reactions and IgE serology to environmental allergens can also be observed in dogs without signs of AD. As a result, these tests cannot be used to differentiate dogs with AD from healthy dogs or dogs with other pruritic dermatoses. Serological and intradermal tests to determine hypersensitivity to food allergens are not recommended to assess the presence of food hypersensitivity in dogs with food-induced AD [1].
  - Updated 2015 recommendations:
    - There is increasing evidence that healthy dogs and/or dogs with pruritic dermatoses other than AD might have detectable serum allergen-specific IgE, and/or positive IDT reactions to environmental allergens, especially those that are not pollens. This reinforces the concept that “allergy tests” must never be used to diagnose AD; they should be requested only to define IgE-mediated hypersensitivities in dogs already diagnosed with AD by clinical criteria (SOR C). There is currently no standardization in the performance of serum allergen-specific IgE assays for environmental allergens, and there is evidence that the results of IgE serological tests can vary substantially between laboratories (SOR C).
    - Because of inconsistent or limited data available, additional studies are needed before recommending the use of specific IgG and IgE serology for, or intradermal or epicutaneous (patch) or lymphocyte stimulation tests with, food allergens to diagnose, or identify relevant food allergens in dogs with food-induced AD (SOR C).
  - Basis for such recommendations:
    - A recent study that compared IgE serological assays at four different laboratories showed a high variation in tests results, except for mite allergens for which there was generally a stronger agreement (QOE 3) [28]. A recent evaluation of an IgG/IgE food allergen serology test (Sensitest, Avacta Veterinary Laboratories) reported that a negative serology result for a food allergen predicted the lack of clinical reaction to this food item in most dogs (negative predictive value of ~80 %); the reverse was not true for dogs with positive serology to food allergens (low positive predictive value) (QOE 2) [29]. Another study from the United Kingdom demonstrated that food-specific IgE/IgG serology offered by two unidentified commercial laboratories did not permit the differentiation of dogs with cutaneous adverse food reactions from those with non-food induced diseases (QOE 2) [30].
    - Patch testing with food items has been shown to have a very high negative predictive value when compared to the response to a restrictive diet trial [29]. Consequently, this method might be useful to identify food items to which dogs are not likely to react clinically.
    - Finally, in a small study from Japan, most dogs with signs of allergic skin disease that had a negative IgE serology to environmental allergens and a positive lymphocyte proliferation test to food allergens had a favourable response to a dietary restriction trial (QOE 3) [31].

- *B.1.d. Implementation of house dust mite control measures*
  - Summary of 2010 guidelines:
    - House dust mites are the most important source of allergens for canine AD, worldwide. House dust mite control measures should be relevant and might be effective in dogs hypersensitive to such allergens. The individual, or combinations of, house dust mite control measures most effective to prevent the flares of dogs with AD still have not been determined [1].
  - Updated 2015 recommendations:
    - There are no major changes to the 2010 recommendations (SOR C).
  - Basis for such recommendations:
    - There is still only one uncontrolled study that reported the benefit of house dust mite control with a benzyl benzoate acaricidal spray (Acarosan Spray, Bissell) for reduction of clinical signs of AD in mite-hypersensitive atopic dogs (QOE 2) [32].
    - Recently, the isolation of dogs with AD in cages in which house dust mites were controlled was shown to lead to a rapid reduction in pruritus in most dogs with IgE hypersensitivity to environmental allergens (QOE 2) [33].

- *B.1.e. Evaluation of use of antimicrobial therapy*

- Summary of the 2010 guidelines:
  - Antimicrobial therapy is needed in an atopic dog when a skin and/or ear infection with bacteria and/or yeast is diagnosed based on compatible clinical signs with or without supportive cytology or bacterial culture. The treatment of such infections usually consists of topical and/or systemic antimicrobials [1].
- Updated 2015 recommendations:
  - There are no major changes to the 2010 recommendations (SOR C). Veterinarians are advised to follow antimicrobial treatment guidelines established in their country of practice and/or in international consensus recommendations (SOR C) [6, 7]. Veterinarians and dog owners should watch for a drying or irritating effect of topical antimicrobials—especially shampoos—that might induce a flare of AD in their patient (SOR C).
  - Terbinafine or itraconazole can be prescribed once daily or for two consecutive days each week for 3 weeks to treat flares provoked or exacerbated by *Malassezia* skin infections (SOR B).
- Basis for such recommendation:
  - Treating dogs with *Malassezia* otitis or dermatitis with 5 mg/kg itraconazole once daily or for two consecutive days each week for 3 weeks, provides comparable clinical and cytological results (QOE 2) [34]. Terbinafine given to dogs with *Malassezia* dermatitis at 30 mg/kg once daily for 3 weeks resulted in a similar improvement in cytological and skin lesion scores as in dogs given the drug at the same dose twice weekly for 3 weeks; the improvement in pruritus was higher with the daily treatment (QOE 2) [35].

- *B.1.f. Investigation of the relevance of other flare factors*
  - Summary of 2010 guidelines:
    - There is insufficient evidence to make general recommendations regarding the importance of the environment, humidity, detergents and stress as flare factors in dogs with AD. Nevertheless, owners should be educated to observe, and then avoid or alter, the specific situations in which they see their dog’s condition worsen [1].
  - Updated 2015 recommendations:
    - There are no changes to the 2010 recommendations (SOR C).

#### B.2. Improvement of skin and coat hygiene and care

- *B.2.a. Bathing with a non-irritating shampoo*
  - Summary of 2010 guidelines:
    - Bathing at least once weekly with a mild non-irritating shampoo and lukewarm water is likely to be beneficial. The intensity and frequency of bathing may be the most important factor in relieving pruritus. The type of shampoo should be tailored to each case: emollient shampoos are likely to be the most soothing, but anti-seborrhoeic and antiseptic products may be more appropriate in dogs with skin greasiness, scaling and/or in case of infection. Nevertheless, shampooing may be drying and irritating. If necessary, clinicians should consider changing products or protocols and/or adding post-bathing topical moisturizers. Practitioners should also be prepared to change the topicals used if the state of the dog’s skin and coat changes. The impact of frequent bathing on the reduction of efficacy of topical flea control products should also be considered [1].
  - Updated 2015 recommendations:
    - There are no changes to the 2010 recommendations (SOR C).

- *B.2.b. Supplementation with oral EFAs*
  - Summary of 2010 guidelines:
    - The oral intake of EFAs, especially those rich in omega-6 EFAs either as supplement or in enriched diets can influence superficial skin lipids and improve the gloss and quality of the coat. Oral EFAs might also provide some small benefit in reducing clinical signs of AD in dogs, but the limited degree of improvement expected makes it unlikely that EFA supplementation would be suitable for monotherapy of canine AD. The benefit of EFAs, if any, might not be seen before two months of supplementation. At this time, there is no evidence of superiority for any particular EFA combination, dosage, ratio or formulation (including enriched diets) to improve skin and coat quality in dogs with AD. In general, EFA-enriched diets provide higher amounts of EFAs than oral administration of EFA supplements [1].
  - Updated 2015 recommendations:
    - There are no changes to the 2010 recommendations (SOR C).
  - Basis for such recommendations:
    - A systematic review did not uncover further clinical trial evidence on the benefit of oral EFAs for canine AD since 2010 (QOE 1) [4]. Supplementing the diet of dogs with AD with an EFA liquid supplement (Megaderm/EFA-Z, Virbac) for two months resulted in marked changes in the biochemistry and ultrastructure of stratum corneum intercellular lipids, with both parameters becoming closer to normal characteristics compared to before supplementation (QOE 3) [36].

- *B.2.c. Application of topical EFA-containing formulations*
  - Summary of the 2010 guidelines:
    - There is insufficient trial-based evidence supporting the use of lipid-containing topical formulations to improve coat quality and/or to relieve signs of AD in dogs [1].
  - Updated 2015 recommendations:
    - Topical lipid formulations can help normalize existing stratum corneum lipid barrier defects in dogs with AD (SOR C). Because of inconsistency in outcomes of clinical trials, there is still insufficient evidence for the benefit of lipid-containing topical formulations to recommend these as monotherapy for canine AD (SOR B). The benefit, cost and ease of use of topical EFA-containing formulations as adjuvant therapy for canine AD must be weighed against those of feeding oral EFA supplements or enriched diets (SOR C). The benefit of topical EFA-containing formulations is likely minimal in dogs already fed EFA-rich diets or EFA supplements (SOR C).
  - Basis for such recommendations:
    - The application of a topical lipid complex containing ceramides, cholesterol and EFAs in a proportion aimed at reproducing that of intercellular stratum corneum lipids (Allerderm Spot On, Virbac) every three days for six applications to atopic dogs normalized pre-existing stratum corneum lipid profile anomalies (QOE 3) [37]. This formulation had previously been shown to increase the formation of normal-appearing intercellular stratum corneum lipid lamellae in some dogs with AD (QOE 3) [38]. However, an RCT in dogs with mild-to-moderate AD only reported a small and inconsistent clinical benefit of this topical lipid complex (QOE 2) [18]. A small RCT established the modest efficacy of an omega-6 EFA and essential oil-containing topical formulation (Dermoscent Essential 6 spot-on, Laboratoire de Dermo-Cosmétique Animale) for reducing clinical signs of AD (QOE 2) [39].
    - As orally administered EFAs can normalize stratum corneum lipid in the same way as a topical lipid mixture (QOE 3) [36–38], the addition of topical EFA-containing formulations to dogs already fed high levels of EFAs is likely to provide little added benefit.

- *B.2.d. Administration of other dietary supplements*
  - Summary of 2010 guidelines:
    - Some nutritional supplements can improve skin barrier function *in vitro*, for example increasing ceramide production and decreasing transepidermal water loss, but there is no evidence for the clinical benefit of such supplements in dogs with AD [1].
  - Updated 2015 recommendations:
    - There are no changes to the 2010 recommendations (SOR C).

#### B.3. Reduction of pruritus and skin lesions with pharmacological agents

- *B.3.a. Treatment with topical glucocorticoids or tacrolimus*
  - Summary of the 2010 guidelines:
    - Topical glucocorticoids and tacrolimus effectively reduce clinical signs of canine AD, but there is a risk of skin atrophy with the prolonged used of the former [1].
  - Updated 2015 guidelines:
    - There is further evidence supporting the efficacy of topical glucocorticoids for treatment of canine AD. However, the risk of induced skin atrophy means that they should be applied intermittently after an induction phase of daily application (SOR A). Treatment duration and frequency of use should be tailored to each patient; the application of topical glucocorticoids should normally continue until a complete and stable remission of signs is achieved (SOR C). Due to its high cost, tacrolimus does not offer much added value compared to topical glucocorticoids, except for atopic dogs in which skin atrophy is visible (SOR C).
  - Basis for such recommendation:
    - In a 12-week RCT, a hydrocortisone aceponate spray (Cortavance, Virbac) showed a similar efficacy and tolerance compared to oral ciclosporin (Atopica, Elanco Animal Health) (QOE 1) [40].

- *B.3.b. Treatment with oral pharmacological immunomodulators*

- Summary of the 2010 guidelines:
  - Oral glucocorticoids and ciclosporin are beneficial for the treatment of canine AD, but the former lead to faster improvement than the latter. Oral short-acting glucocorticoids should be used to induce remission of signs, and their dose should then be tapered; injectable long-lasting glucocorticoids are not recommended. The long-term concurrent administration of oral ciclosporin and glucocorticoids (especially at higher dosages of either or both drugs) is likely to result in a higher risk of immunosuppression [1].
- Updated 2015 recommendations:
  - Oral glucocorticoids (prednisone, prednisolone or methylprednisolone), ciclosporin and oclacitinib are effective for treatment of chronic canine AD (SOR A), concurrently with or after control of known flare factors (SOR C). Glucocorticoids and oclacitinib lead to faster improvement than ciclosporin, but ciclosporin can be combined with oral prednisolone for the first 3 weeks to speed its onset of clinical improvement (SOR A). The prolonged concomitant administration of oral glucocorticoids, ciclosporin or oclacitinib in any combination is not recommended because of the theoretical higher risk of immunosuppression predisposing to potentially severe opportunistic infections of the skin or other organs. There is no consensus on the need for laboratory monitoring (e.g. haematology, serum biochemistry and urinalysis) during prolonged ciclosporin or oclacitinib administration. However, such tests should be performed if signs of systemic illness develop (SOR C). Due to the increased risk of urinary tract infections, dogs treated with oral glucocorticoids in the long term should be monitored periodically with urinalyses and urine cultures (SOR C).
  - Oral glucocorticoids (prednisolone, prednisone or methylprednisolone) should be used at 0.5 mg/kg once to twice daily to induce remission of clinical signs of AD. After such remission occurs, the dose of oral glucocorticoids should be tapered to the lowest dosage and frequency that maintains an absence of signs to minimize the risk of side effects in the long term (SOR C). Long acting injectable glucocorticoids should be avoided wherever possible as the lack of ability to taper their dose increases the risk of adverse events (SOR C).
  - Oral ciclosporin should be administered at 5 mg/kg once daily until satisfactory control of clinical signs, which will usually take 4 to 6 weeks (SOR A). Thereafter, the dose required to maintain remission should be tapered by either decreasing the frequency of treatment (e.g. from every day to every other day and then twice weekly) or by decreasing the daily dose (SOR A). Generic ciclosporin formulations shown to be bioequivalent to the first approved ciclosporin (modified) microemulsion (Atopica, Elanco Animal Health) are acceptable substitutes for it (SOR C).
  - Oral oclacitinib (Apoquel, Zoetis) should be given at 0.4 to 0.6 mg/kg twice daily for 14 days and then once daily thereafter (SOR A). In case a complete remission of signs is obtained, further tapering should be attempted with the dose adjusted to maintain the remission of signs (SOR C). This drug is not approved for dogs less than 12 months of age. The long-term administration of oclacitinib administered once daily appears to be relatively safe whereas the long-term safety of other dosing regimens is not known.
  - The concomitant use of allergen-specific immunotherapy, emollient shampoos, EFAs supplements or enriched diets might allow for a further reduction in the dose and/or frequency of oral glucocorticoids, ciclosporin (and perhaps even oclacitinib) required to maintain remission of clinical signs of AD. Outside of the oral glucocorticoid-sparing effect of an EFA supplement (Viacutan Plus, Boehringer Ingelheim) and an antihistamine (trimeprazine)-prednisolone combination (Temaril-P, Zoetis), which were both discussed in the 2010 version of these guidelines [1], the efficacy and safety of other combined approaches has not yet been published (SOR C).
- Basis for such recommendations:
  - Three systematic reviews, as well as newly-published randomized controlled trials, have confirmed the efficacy of oral glucocorticoids [2–4, 11, 12], ciclosporin [3, 4, 13, 41, 42] and oclacitinib [11, 42, 43] for treatment of AD in dogs (QOE 1). Further details on the treatment of chronic canine AD with oral glucocorticoids and ciclosporin can be found in the 2010 guidelines [1].
  - In a RCT, ciclosporin orally given at 5 mg/kg daily for 4 weeks, concurrently with prednisolone at 1 mg/kg daily for 7 days followed by alternate day dosing for 14 days, led to a quicker improvement of skin lesions and pruritus scores than when ciclosporin was given alone (QOE 2) [44]. A generic formulation of ciclosporin (Equoral, Teva) was shown to be as effective as prednisone in reducing skin lesions and pruritus in dogs with AD in a small RCT (QOE 2) [13]. A novel liquid oral formulation of ciclosporin (Cyclavance, Virbac) was recently reported to be better accepted than ciclosporin capsules (Atopica, Elanco Animal Health) (QOE 2) [45].
  - In RCTs, oclacitinib improved pruritus and clinical signs significantly better than placebo (QOE 1) [43], and as well or (at the 14 day time point) better than prednisolone (QOE 1) [11]. The long term administration of oclacinitib is associated with the development of *de novo* urinary tract infections, vomiting, otitis, pyoderma and diarrhoea in approximately 5 to 10 % of dogs; serious adverse drug events appear rare (QOE 1) [46]. Changes in laboratory (haematology, chemistry panels and urinalysis) parameters seem minimal after the prolonged administration of oclacitinib to atopic dogs (QOE 1) [46].

- *B.3.c. Treatment with biotherapeutic immunomodulators*
  - *B.3.c.1 Treatment with recombinant interferons*

- Summary of the 2010 guidelines:
  - Recombinant canine interferon-gamma, given subcutaneously at 5,000–10,000 units/kg three times weekly for 4 weeks, then once weekly, is effective for treatment of canine AD. Recombinant feline interferon-omega seems to be beneficial, but further trials are warranted before recommending its use [1].
- Updated 2015 recommendations:
  - Recombinant canine interferon-gamma (Interdog, Toray Industries), given subcutaneously at 5,000–10,000 units/kg three times weekly for 4 weeks, then once weekly, is effective for treatment of canine AD (SOR A). Recombinant feline interferon-omega (Virbagen omega, Virbac), administered subcutaneously or orally, has been shown to provide some inconsistent reduction of skin lesions and pruritus in dogs with AD (SOR B).
- Basis for such recommendations:
  - Two RCTs provided evidence for the efficacy of recombinant canine gamma-interferon (Interdog, Toray Industries) to treat dogs with AD in Japan (QOE 1) [47, 48]; suggested effective dosages are 5,000 to 10,000 units/kg subcutaneously three times weekly for 4 weeks then once weekly. Side effects appear to be minimal [47, 48].
  - Results of two studies, including one RCT, established that subcutaneous injections of recombinant feline interferon-omega (Virbagen Omega, Virbac), at 1 to 5 million units three times weekly for 4 weeks and then monthly thereafter, offer some clinical benefit in dogs with AD (QOE 1) [49]. Another RCT showed some inconsistent and mild improvement in skin lesions and pruritus after either subcutaneous injections or oral administration of feline interferon-omega (QOE 2) [50].

- *B.3.d. Interventions likely to be of little or no benefit to treat chronic canine AD*:
  - Summary of the 2010 guidelines:
    - There is a lack of evidence for efficacy of type 1 antihistamines as monotherapy for the management of chronic canine AD. Hydroxyzine and its metabolite cetirizine have demonstrable anti-histaminic action in the dog and should be the preferable antihistamine in this species. Antihistamines should be used as preventatives, given on a continuous daily basis, and a combination with other antihistamines or other drugs may improve their beneficial effects although further studies are required to validate this. Other drugs appear to provide little (misoprostol, tepoxalin) or no benefit (e.g. leukotriene inhibitors, capsaicin, dextromethorphan etc.) [1].
  - Updated 2015 recommendations:
    - Type 1 histamine receptor inverse agonists (type 1 antihistamines) have modest efficacy against pruritus, either alone or in combination with each other, but their effect appears to be variable between individuals. For optimal efficacy, this class of drugs are best used as preventatives before a flare occurs—not during or after it—and they should preferably be given on a continuous daily basis. In dogs, antihistamines with proven bioavailability and/or demonstrated reliable efficacy in this species should be the preferred choices (SOR C).
    - Masitinib (Masivet/Kinavet, AB Science) appears to offer some benefit in dogs with chronic AD, but this effect must be weighed against the risk of severe renal adverse drug events that requires the performance of periodic urinalyses to detect developing proteinuria (SOR A). Masitinib might be a useful alternative for atopic dogs with signs not responding to other approved drugs (SOR C).
    - Further studies are needed to confirm the efficacy and safety of high-dose oral pentoxifylline, oral low-dose once weekly methotrexate and the adjunctive effect of vitamin E to antihistamines before these drugs can be recommended for routine treatment of AD in dogs (SOR C). Finally, oral fluoxetine and low level laser therapy appear to have little efficacy for treatment of canine AD (SOR B).
  - Basis for such recommendations:
    - An RCT evaluating the efficacy of the antihistamines dimetindene (Fenistil, Novartis) and the combination of chlorpheniramine and hydroxyzine (Histacalmine, Virbac) confirmed the small but variable efficacy of H1 antihistamines to control pruritus in dogs with AD. The combination of the two antihistamines did not show any added benefit over the single drug, but this observation cannot be extrapolated to other combinations of drugs from this class (QOE 2) [16]. One small trial suggested a possible benefit from the H1 antihistamine fexofenadine with a reported efficacy similar to that of methylprednisolone (QOE 2) [51]. In another study, dogs were treated with fexofenadine and oral vitamin E or placebo for 8 weeks. An improvement in skin lesions was seen in dogs from both groups with a greater improvement in dogs receiving vitamin E; there was considerable individual response within groups, however (QOE 2) [52].
    - A large RCT confirmed that masitinib at 12.5 mg/kg once daily was moderately effective in reducing clinical signs in atopic dogs. The development of a protein-losing nephropathy in some dogs, which, if unrecognized could be potentially fatal, is a limitation of masitinib treatment (QOE 1) [53].
    - An open RCT study evaluating pentoxifylline at the high dose of 20 mg/kg three times daily, either alone or in combination with oral EFA supplementation, reported a significantly greater improvement in skin lesions and pruritus of these interventions over placebo; the effect seemed highest for dogs treated with the combination of pentoxifylline and EFAs (QOE 2) [54].
    - A small proof-of-concept trial reported the clinical benefit and relative safety of low-dose once weekly oral methotrexate for treatment of canine AD [55].
    - An RCT showed no benefit of low level laser therapy in dogs with localized pedal AD (QOE 2) [56]. Similarly, the selective serotonin reuptake inhibitor (SSRI) fluoxetine, given at 1 mg/kg orally once daily, showed no clinical efficacy in a small RCT of dogs with AD (QOE 2) [57].

#### C. Implement strategies to prevent recurrence of signs

This section is relevant to the treatment of dogs with case scenarios 2a and 2b described in the 2010 version of these guidelines [1].

- *C.1. Avoidance of flare factors*
  - Summary of the 2010 guidelines:
    - The identification and avoidance of known flare factors (e.g. environmental and/or food allergens, flea bites, infections etc.) is the best strategy to prevent the recurrence of signs in patients with AD [1].
  - Updated 2015 recommendations:
    - There are no proposed changes to the 2010 recommendations (SOR C).

- C.2. *Implementation of proactive topical pharmacotherapy*
  - Summary of the 2010 guidelines:
    - In humans with AD, there is evidence for the high benefit, cost effectiveness and low risk of proactive intermittent applications of topical glucocorticoids and tacrolimus to previously affected skin areas to delay or prevent the appearance of such flares. There is currently no evidence for the effectiveness of a similar approach in dogs with AD, but the possible benefit, low risk and low cost suggest that such strategy is worth considering in suitable cases [1].
  - Updated 2015 guidelines:
    - The application of a topical hydrocortisone aceponate spray (Cortavance, Virbac) to areas of previous skin lesions, two consecutive days each week, can delay the recurrence of lesions at these sites without causing visible skin atrophy (SOR B). A similar beneficial effect of proactive topical glucocorticoid therapy is likely to be seen when intermittently using other moderately potent topical glucocorticoids at previously affected skin sites (SOR C). When using potent topical glucocorticoid formulations, even intermittently, care must be taken to avoid glucocorticoid-induced skin atrophy (SOR C).
  - Basis for such recommendation:
    - A small RCT tested the efficacy a hydrocortisone aceponate spray (Cortavance, Virbac) applied to previously affected areas on two consecutive days after lesions had been controlled with the same spray. The time to recurrence of flares at these sites was nearly four times longer (median: 115 days) in dogs intermittently-treated with topical glucocorticoids compared to those sprayed with placebo (QOE 2) [58].

- *C3. Implementation of allergen-specific immunotherapy*
  - Summary of the 2010 guidelines:
    - Allergen-specific immunotherapy (ASIT) is an effective and safe way to reduce the clinical signs of AD in dogs. There is no proven superiority of a particular ASIT protocol over other alternatives (traditional, rush or low-dose). Injection frequencies and amounts injected must be tailored to each patient depending upon the clinical improvement observed and the presence of adverse events. Because of the delay in the onset its beneficial effects, anti-inflammatory drugs should be given temporarily, as needed to maintain good quality of life, until such time as the ASIT is judged to be effective (see sections above). Because the onset of clinical benefit might not appear for months, ASIT must be continued for at least one year to properly evaluate its efficacy. Whether or not ASIT must be continued for the reminder of the life of atopic dogs has not been established [1].
  - Updated 2015 recommendations:
    - The value of ASIT as a canine AD-modifying treatment continues to be supported by (mostly uncontrolled) studies reporting at least a moderate efficacy (SOR B). There is some evidence that ASIT administered via the sublingual route (sublingual immunotherapy; SLIT), or in sped-up (i.e. “rush”) protocol, are safe and effective for treatment of atopic dogs (SOR C). While most patients appear to require many years of ASIT, attempts should be made to decrease the frequency of administration, or even stop this intervention, in dogs exhibiting a prolonged complete remission of signs (SOR C).
    - There is currently no standardization in the performance of allergen-specific intradermal tests or IgE serologies that are used used to select allergens to be included in ASIT. Mounting evidence suggests that the results of serological tests can vary substantially between laboratories (SOR C). A consequence of such assay variability is that recommendations for immunotherapy prescriptions are expected to vary substantially between testing laboratories (SOR C).
  - Basis for such recommendation:
    - A recent study comparing IgE serological assays offered by four different laboratories showed a substantial variation in both results and subsequent ASIT recommendations (QOE 3) [28]. Similarly, intradermal testing for allergens is not standardized and its performance varies substantially even between specialists of the same geographical region [59].
    - In spite of these important limitations in allergen hypersensitivity tests, an online survey showed that one third of owners of atopic dogs who had used this intervention for 5 to 10 years rated it as “very or extremely effective” (QOE 2) [15]. Moreover, approximately 5 % of dogs having received ASIT as part of their treatment had an apparent complete resolution of signs without further need of anti-allergic treatment [15]. Similarly, a large retrospective survey of owners of atopic dogs having undergone 1 year or more of ASIT established that nearly two third of dogs had been rated as having a “satisfactory-to-excellent” response to this intervention (QOE 2) [60].
    - A small, open pilot study of SLIT in house dust mite-sensitive atopic dogs reported clinical improvement and changes in mite-specific IgG and IgE in most dogs (QOE 2) [61]. Similarly, a larger, retrospective study of SLIT in house dust mite and pollen-hypersensitive dogs reported a good-to-excellent response to SLIT in about 60 % of evaluable dogs, and in half of those who had failed previous subcutaneous ASIT (QOE 2) [62].
    - Finally, in a small open study of rush alum-adjuvanted ASIT, atopic dogs demonstrated a significant improvement in pruritus and medication scores after one year of treatment (QOE 2) [63].

- *C4. Implementation of nonspecific immunotherapy*
  - Summary of the 2010 guidelines:
    - This is a new section that was not included in the 2010 guidelines [1].
  - Updated 2015 recommendations:
    - There is currently insufficient evidence supporting the use of oral probiotics as nonspecific immunotherapy for prevention or treatment of canine AD (SOR C).
  - Basis for such recommendation:
    - Even though the pre- and post-natal exposure to the probiotic *Lactobacillus rhamnosus* strain GG (Culturelle HS, Culturelle) has shown some possible lasting effect in reducing clinical signs following allergen challenges in dogs experimentally sensitized to house dust mites (QOE 3) [64], this oral probiotic has not yet been shown to be of benefit in dogs to treat or prevent clinical signs in dogs with spontaneous AD.

## Conclusion

This first 5-year minor update of the international consensus guidelines for treatment of AD further highlights, as was done with the first version of this document [1], that the treatment of this disease is clearly multifaceted and that interventions should be combined for a proven (or likely) optimal benefit. Furthermore, treatment should be tailored to each patient depending upon the stage of the disease, its severity and the distribution of lesions. Veterinarians should also remember to evaluate and then discuss with the pet owners the benefit of each recommended intervention, its side effects, its ease of administration, and its cost as a single or combined modality. Ultimately, the quality of life of both dogs and their owners, as well as the preferences of the latter, should be considered before a treatment plan is designed.

## Additional file

Additional file 1:
**Treatment of canine atopic dermatitis: summary statement of 2015 ICADA guidelines. **(DOCX 148 kb)

## Abbreviations

AD: Atopic dermatitis
RCT: Randomized controlled trial
