# Supplementary material for: Treatment of canine atopic dermatitis: 2015 updated guidelines from the International Committee on Allergic Diseases of Animals (ICADA)
Source: BMC Vet Res. 2015 Aug 16;11:210. doi: 10.1186/s12917-015-0514-6 (PMC4537558; doi:10.1186/s12917-015-0514-6)
Supplement: Additional file 1: — Treatment of canine atopic dermatitis: summary statement of 2015 ICADA guidelines. (DOCX 148 kb) [file 12917_2015_514_MOESM1_ESM.docx]

Treatment of Canine Atopic Dermatitis:

Summary Statement of 2015 Guidelines

*Preamble*: it is unlikely that a single intervention will be effective to treat AD in every dog. Veterinarians should consider combining interventions to maximize benefit while minimizing cost and adverse drug events.

1. **Treatment of acute flares of canine atopic dermatitis:**
   1. *Identification and avoidance of flare factors:*
      1. Identification and elimination, whenever possible, of allergenic flare factors (fleas, food and environmental allergens)
      2. Evaluation of use of antimicrobial therapy if clinical signs of infection with bacteria or yeast are present on the skin or in the ears
   2. *Improvement in skin and coat hygiene and care:*
      1. Bathing with a non-irritating shampoo
   3. *Reduction of pruritus and skin lesions with pharmacological agents:*
      1. Treatment with topical glucocorticoids, especially for localized lesions, as needed to control signs
      2. Treatment with oral glucocorticoids or oclacitinib, especially for widespread or severe lesions, as needed to control signs
2. **Treatment of chronic canine atopic dermatitis:**
   1. *Identification and avoidance of flare factors:*
      1. Dietary restriction-provocation trials in dogs with nonseasonal signs
      2. Implementation of an effective flea control regimen in areas where fleas are present
      3. Performance of allergen-specific intradermal and/or IgE serological tests to identify possible environmental allergen flare factors
      4. Possible implementation of house dust mite or other allergen control measures, if relevant and feasible
      5. Evaluation of use of antimicrobial therapy if signs of infection or colonization with bacteria or yeast are present on the skin or in the ears
   2. *Improvement in skin and coat hygiene and care:*
      1. Bathing with a non-irritating shampoo or an antiseborrheic/antimicrobial shampoo, depending on the skin lesions seen
      2. Dietary supplementation with essential fatty acids
   3. *Reduction of pruritus and skin lesions with pharmacological or biological agents:*
      1. Treatment with topical glucocorticoids especially for localized lesions, as needed to control signs
      2. Treatment with oral glucocorticoids, ciclosporin, oclacitinib or injectable interferons (where available), especially for widespread or severe lesions, as needed to control signs. These drugs should not be combined together in the long term to reduce the risk of immunosuppression
      3. Trial of EFAs and antihistamines as glucocorticoid-sparing agents if these are being used in the long term
   4. *Implementation of strategies to prevent the recurrence of signs*
      1. Avoidance of known flare factors, as identified above
      2. Consideration of proactive intermittent topical glucocorticoid therapy, if feasible and relevant
      3. Implementation of allergen-specific immunotherapy, if feasible. This can be used alongside all the above treatment options in an attempt to provide a long-term amelioration of the aberrant immune response.
